# Evaluation of the proteomic profiles of ejaculated spermatozoa from Saanen bucks ( *Capra hircus* )

**DOI:** 10.21451/1984-3143-AR2019-0001

**Published:** 2019-11-18

**Authors:** Tatiana Maria Farias Pinto, Raulzito Fernandes Moreira, Maria Nagila Carneiro Matos, Vitória Virginia Magalhães Soares, Mônica Valeria de Almeida Aguiar, Paulo de Tarso Teles Dourado de Aragão, João Garcia Alves, Frederico Bruno Mendes Batista Moreno, Ana Cristina de Oliveira Monteiro-Moreira, Cíntia Renata Rocha Costa, José Luiz de Lima, Angela Maria Xavier Eloy, Rodrigo Maranguape Silva da Cunha

**Affiliations:** 1 Universidade Estadual Vale do Acaraú Laboratorio de Biologia Molecular Sobral Brasil Universidade Estadual Vale do Acaraú, Laboratorio de Biologia Molecular, Sobral, Brasil; 2 Universidade de Fortaleza Núcleo de Biologia Experimental Fortaleza Brasil Universidade de Fortaleza, Núcleo de Biologia Experimental, Fortaleza, Brasil; 3 Universidade Federal de Pernambuco Federal Departamento de Bioquímica Laboratório de Imunopatologia Keizo Asami Recife Brasil Universidade Federal de Pernambuco Federal, Departamento de Bioquímica, Laboratório de Imunopatologia Keizo Asami, Recife, Brasil; 4 Centro de Pesquisa Caprinos e Ovinos Empresa Brasileira de Pesquisa Agropecuária Sobral Brasil Centro de Pesquisa Caprinos e Ovinos, Empresa Brasileira de Pesquisa Agropecuária, Sobral, Brasil

**Keywords:** saanen, sperm, proteomic profiles

## Abstract

The Saanen goat breed has been widely explored in breeding programmes; however, there are few reports about the breed’s genetic and molecular composition. Thus, this study aimed to characterize the proteomic profile of spermatozoa from Saanen breeding goats. Five breeding animals with proven fertility were selected, the spermatozoa were collected, and the protein was extracted. Subsequently, the proteins were separated and analysed by two-dimensional electrophoresis and mass spectrometry; the proteins were then identified with the SwissProt database. A total of 31 proteins involved in reproduction were identified, including binding proteins on spermatozoa for fusion with the egg, acrosomal membrane proteins, metabolic enzymes, heat shock proteins, cytoskeletal proteins and spermatozoa motility proteins. The characterization of such proteins clarifies the molecular mechanisms of spermatogenesis and the modifications that ensure the success of fertilization.

## Introduction

The Saanen breed was introduced to Brazil because it presents high production rates that have been explored with genetic crosses. However, molecular reports for this breed in the environmental conditions of Northeast Brazil are scarce; this is mainly true for the males since they contribute significantly to the genetics of the herd ( Lôbo and Silva 2008 ).

The understanding of the process of male gamete formation and the search for fertility markers are great challenges for modern animal livestock production, and proteomic studies can provide and reveal answers to such questions ( Brewis and Gadella, 2010 ; Peddinti et al., 2008 ). Spermatozoa are transcriptional and translationally silent, and the proteomic approach to study sperm function is essential ( Saraswat et al., 2017 ).

Proteomic studies have provided a better understanding of the protein function in sperm processes and in different functional stages of sperm. These studies demonstrate the importance of post-translational modifications (phosphorylation, glycosylation, acetylation, and proteolytic cleavage) in the physiology of sperm function. This information is fundamental for the discovery of new male fertility biomarkers that may allow a better diagnosis of sperm dysfunction and therapeutic intervention ( du Plessis et al., 2011 ; Nixon et al., 2010 ; Baker, 2016 ). Comparative analyses employing proteomics techniques have also allowed the identification of proteins of interest in fertile breeding animals compared to the protein profiles of infertile animals ( Peddinti et al., 2008 ; Oliva et al., 2010 ).

The new advances in proteomics may also contribute to the development of new approaches to regulate fertility, to understand the causes of male infertility and to enable biotechniques in mammals, such as in vitro fertilization ( Aitken and Baker, 2008 ; Bilic et al., 2018 ). Thus, the objective of this study was to establish the profile of goat spermatozoa of the Saanen breed and their roles in reproductive development.

## Methods

### Chemicals

Acrylamide, bisacrylamide, Dithiothreitol (DTT), iodoacetamide, 3-[(3-Cholamidopropyl)dimethylammonio]-1-propanesulfonate (CHAPS), Sodium Dodecyl Sulfate (SDS), urea, glycerol, thiourea, Tetramethylethylenediamine (TEMED), Ammonium Persulfate (APS), molecular markers and Immobilized pH Gradient (IPG) buffer were obtained from GE Healthcare Life Sciences (São Paulo, SP, Brazil). Triton X-100, Bovine Serum Albumin (BSA) and Coomassie Brilliant Blue (CBB) were obtained from Sigma-Aldrich (São Paulo, SP, Brazil). Trypsin was obtained from Promega (São Paulo, SP, Brazil).

### Experimental animals and semen collection

Research was approved by the Research Ethics Committee, approval number 001.04.013.UVA.505.01. Five healthy male goats ( *Capra hircus* ) of the Saanen breed weighing 82.6 ± 3.4 kg and aged from 18 to 21 months were provided by the experimental farm of Embrapa Caprinos and Ovinos from the city of Sobral - Ceará; this is a semi-arid region of Northeast Brazil located at 03° 44' south latitude and 40° 20' west longitude with an altitude of 109.62 metres, maximum and minimum average temperatures of 33.9 °C and 23.1 °C, respectively, and a relative humidity of 70% (data were obtained from the National Institute of Meteorology; INMET, 2019 ). During the subsequent experiments, the animals were subjected to a controlled diet, receiving elephant grass ( *Pennisetum purpureum* ) supplemented with 300 g of concentrate per day, containing 70% corn, 27% soybean meal, 2% limestone and 1% mineral salt. Semen collection was performed using an artificial vagina and an ovariectomized female whose oestrus cycle was induced using 1 mL of oestradiol cypionate. The samples were collected once per week in the months of March and April of 2013 between 08:00 a.m. to 10:00 p.m., totalling 8 collections per animal.

### Protein extraction and measurement

The extraction of the total proteins was performed as described by Moreira et al. (2017) . The eight semen samples collected per animal were pooled. The samples were centrifuged at 1,500 x g for 30 minutes at 5 °C to separate the seminal plasma and spermatozoa. The spermatozoa were then washed with a phosphate-buffered saline solution (PBS, pH 7.4) and were centrifuged three times at 4,000 x g for 10 minutes at 4 °C. Aliquots of cells were separated for extraction using 4% CHAPS detergent, 7 M urea, 2 M thiourea, and 20 mM DTT. The samples were added to 300 µL of extraction buffer and stirred for two hours on ice. The samples were then centrifuged at 10,000 x g for 20 minutes at 4 °C, and the supernatants were stored.

The proteins were quantified using the Bradford method ( Bradford, 1976 ), and the protein quality was analysed using SDS-PAGE ( Laemmli, 1970 ).

### Two-dimensional electrophoresis

The gels were made in triplicate per animal, totalling 15 profile maps. Spermatozoan proteins (250 µg) were solubilized in rehydration buffer (7 M urea, 2 M thiourea, 65 mM DTT, 1% (w/v) CHAPS, 0.5% (v/v) ampholytes, and trace amounts of bromophenol blue. The samples were applied to an IPGBox (GE Healthcare) and were incubated on 13 cm IPG strips with a linear pH gradient (pH 4-7) for 16 hours.

Isoelectric focusing was performed using an Ettan™ IPGPhor 3™ Focusing Unit (GE Healthcare) under the following conditions: step 1, 500 V for 30 minutes; step 2, 4,000 V for 2.5 hours; and step 3, 8,000 V until 18,000 total volt-hours is reached. The strips were then stored at -80 °C for later use. The strips were equilibrated in an equilibrium solution (50 mM Tris, 30% glycerol, 6 M urea, 2% SDS and trace amounts of bromophenol blue) with 1% (w/v) DTT for 15 minutes. Strips were then immediately incubated in an equilibrium solution containing 3% (w/v) iodoacetamide for 15 minutes. Finally, the proteins were separated along the second dimension using 12.5% polyacrylamide gels in the presence of SDS with 15 mA/gel for 15 minutes and 50 mA/gel for 4-8 hours.

### Protein staining and analysis

The proteins were stained with CBB G-250 solution (Blue Silver) as previously described ( Candiano et al., 2004 ). An ImageScanner III was used to digitize the gels, and the images were managed using LabScan 6.0 software (both from GE Healthcare). The images were analysed using ImageMaster 2D Platinum 6.0 software (GE Healthcare). The spots were analysed based on their area, volume and intensity, as well as distribution similarity among the triplicates. The student's t test was used, performed automatically by the software.

### Mass spectrometry

The size of the analysed spots ranged from approximately 1 mm to 2 mm, larger spots were subdivided with reference to this margin, the analyses were performed in duplicates. The treated spots were digested with trypsin. The digestions were performed in 50 mM ammonium bicarbonate at 1:50 w/w (enzyme/substrate). All digestions were maintained for 18 hours and were then stopped with 2 μL of 2% formic acid. The peptides were extracted from the gel according to the method described by Shevchenko et al. (2006) .

The digested samples were injected using a nanoAcquity UPLC sample manager, and chromatographic separation was performed using a UPLC C18 column (75 µm x 10 cm) with a 0.35 µL/min flow rate. The mass spectra were acquired using a Synapt G1 HDMS Acquity UPLC instrument (Waters Co., Milford, MA, USA) using data-dependent acquisition (DDA), wherein the three top peaks were subjected to MS/MS. The data were processed using Protein Lynx Global Server software (Waters Co., USA) and were used for a database search using the Mascot search engine ( Perkins et al., 1999 ). The searches were performed by assuming a maximum of one missed trypsin cleavage, mono-isotopic peptides, partially oxidized methionine residues, and fully carbamidomethylated cysteine residues. The peptide masses and fragment mass tolerances were initially set to ± 0.1 Da for MS/MS ion searching; however, candidate peptide IDs were only accepted if the *m/z* values observed were within 0.1 Da (typically less than 0.05 Da) of the theoretical mass of the candidate ID as determined by a manual review of the MASCOT search results.

### Bioinformatics analysis

The enrichment analyses of the Gene Ontology terms ( Ashburner et al., 2000 ) and KEGG pathway ( Kanehisa et al., 2016 ) were performed using the information deposited for *Bos taurus* in both databases and were considered for subsequent analyses only if the terms and pathways had false discovery rate (FDR) values ≤ 0.05 based on the *Bonferroni test* of *p values* obtained by *Fisher's exact test* .

The protein-protein interaction network approach (interactome) was used to analyse the interaction events based on the proteins identified by MS, with reference to the deposited information *Bos taurus* species in the database STRING version 10 ( www.string-db.org ) ( Szklarczyk et al., 2015 ).

The databases were obtained using the STRINGdb package of the R ( Franceschini et al., 2013 ). The regulatory networks were reconstructed using the RedeR package ( Castro et al., 2012 ).

### Statistical analysis

For bidimensional electrophoresis, a comparison between the animals and their experimental replicates was performed. The similarity of the gels was compared by Pearson’s correlation, and the co-efficiency was based on the percentage of spot volume in the gels. The enrichment analyses and interactome were performed for identified proteins using FDR ( *Fisher's exact test* followed by *Bonferroni test* of *p values* ) and confidence, respectively.

## Results

All the extracted proteins showed quality and viable concentrations for further analysis ( Annex 1 and 2 ). Two-dimensional electrophoresis analysis demonstrated distinct protein spots in the reference gels from each animal, and their distribution showed a majority abundance of proteins in the pI ranging from 5 to 6.69 and a molecular weight between 20 and 80 kDa ( Annex 3 ). The Pearson correlation analysis between the reference gel and their duplicates showed such values of 0.915 and 0.964, indicating statistical similarity and, therefore, the reliability of the data.

**Figure 1:**
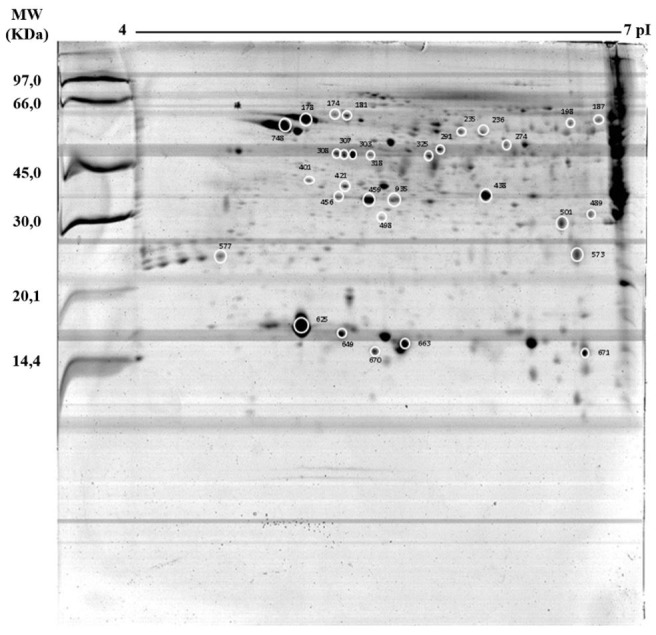
The reference proteomic map (animal C) and proteins identified by MS are indicated by circles. The distribution of the spots ranged in pHs of 4-7 and molecular masses of 14,4-97,0 kDa. The numerals indicate the number of spots and are shown in Table 1 .

Aiming the identification by mass spectrometry, 119 spots were selected according to the intensity and percentage of the volume and analysed by ESI-QUAD-TOF. Among these spots, 31 showed similarity to specific proteins involved with reproduction processes, corresponding to twenty binding proteins, four with catalytic activity and seven related to cellular regulation ( Table 1 and Figure 1 ).

**Table 1:** Identification of Saanen’s sperm proteins after two-dimensional gel separation and the identification of the peptide sequences by ESI-QUAD-TOF mass spectrometry.

**Spot number**	**Protein name**	**Symbol**	**Score**	**MW/pI**	**Sequence Converage (%)**	**Matched peptides**
174	60 kDa heat shock protein, mitochondrial	HSPD1	159	61122/5.83	11	ALMLQGVDLLADAVAVTMGPKISSVQSIVPALEIANA HRVGLQVVAVKAAVEEGIVLGGGCALLR
181	-	-	326	61088/5.91	20	ALMLQGVDLLADAVAVTMGPKLVQDVANNTNEEAGDGTTT ATVLARGYISPYFINTSKISSVQSIVPALEIANAHRVGLQVVA VIQEITEQLDITTSEYEKEKNAGVEGSLIVEK
178	Tubulin alpha-3C/D chain	TUBA3D	1030	50612/4.97	51	AVFVDLEPTVVDEVRQLFHPEQLITGKEDAANNYAREIVDLVLDRNLDIE RPTYTNLNRLIGQIVSSITASLRFDGALNVDLTEFQTNLVPYPRIHFPL ATYAPVISAEKAYHEQLSVAED ITNACFEPANQMVKYMACCML YRDVNAAIATIKT IQFVDWCPTG FKVGINYQPPTVVPGGDLAKAVC MLSNTTAIAEAWARLDHKFDLMYAKAFVHWYVGEGMEEGEFSEAR
274	-	-	477	50612/4.97	27	AVFVDLEPTVVDEVREIVDLVLDRNLDIERPTYTNLNRLIGQIVSSI TASLRIHFPLATYAPVISAEKYMACCMLYRDVNAAIATIKTIQFV DWCPTGFKVGINYQPPTVVPGGDLAKFDLMYAK
401	Tubulin beta chain	TBB	185	38554/5.85	11	GHYTEGAELVDSVLDVVRIMNTFSVVPSPKFPGQLNADLR
498	-	-	203	38554/5.85	10	IM NTFSVVPSPKFPG QLNADLRLH FFMPGFAPLTSR
577	-	-	87	50285/4.78	6	YLTVAAVFREVDEQMLNVQNKISEQFTAMFR
421	Tubulin beta chain	TBB	315	50475/4.73	15	AVLVDLEPGTMDSVRGHYTEGAELVDSVLDVVRIMNTFSVVPS PKFPGQLNADLRLHFFMPGFAPLTSR
748	Tubulin beta-4B chain	TUBB4B	1340	50255/4.79	61	MREIVHLQAGQCGNQIGAKINVY YNEA TGGKAVLVDLEPGTMDSVRSGPFGQIFRPDNFVFGQSGAG NNWAKEAESCDCLQGFQLTHSLGGGTGSG MGTLLISKIREEYPDRIMNTFSVVPSPKLTTPTYGDLNHL VSATMSGVTTCLRFPGQLNADLRKLAVNMVPF PRLHFFMPGFAPLTSRALTVPELTQQMFDAKYLTVAAVFR MSMKEVDEQMLNVQNKNSSYFVEWIPNNVKT AVCDIPPRMSATFIGNSTAIQELFKRISEQFTAMFR
187	Dihydrolipoyl dehydrogenase, mitochondrial	DLD	412	54713/7.95	20	NETLGGTCLNVGCIPSKALTGGIAHLFKIDVSIEAASGGKIP NIYAIGDVVAGPMLAHKSEEQLKEEGIEYKFPFA ANSRVCHAHPTLSEAFREANLAASFGK
198	-	-	318	54713/7.95	16	ALTGGIAHLFKIDVSIEAASGGKIPNIYAIGDV VAGPMLAHKSEEQLKEEGIEYKFPFAANS RVCHAHPTLSEAFREANLAASFGK
235	Outer dense fiber protein 2	ODF2	630	76249/7.52	17	LSTFEETNRLMEQQGTLLKRAEVEAIM EQLKVTDLVNQQQTLEEKASFAPMEDKLNQAH IEVQQLKNYEGMIDNYKTRLEADEVAAQLERL AECQDQLQGYERKNIDLTAIISDLR
236	-	-	242	76249/7.52	14	LMEQQGTLLKVTDLVNQQQTLEEKASF APMEDKLNQAHIEVQQLKNYEGMIDNY KTRLEADEVAAQLERLAECQDQLQGY ERKNIDLTAIISDLR
325	Outer dense fiber protein 2	ODF2	348	76249/7.52	9	AEVEAIMEQLKDLYVAEALSTLESWRNY EGMIDNYKLEADEVAAQLERKNIDLTAIISDLR
291	Actin-like protein 9	ACTL9	494	45993/5.84	42	TGAVVIDMGTGT CKDHPLLFSDPPFSP STNRLVEVAFESLSSPAMYVASQSVLSVYAHG RLDLAGTHLTA FLAEMLLGSG LPLGQQDLDTVENIKYCYV APDFLKRQTLKLPDGRELFQCPELLFSPPEIPGL SPVGVPTMAQ QSLSKFQTELLRTFQSCWVLREQYEEQGPYI VYR
307	Actin, cytoplasmic type 5	ACTG1	505	42151/5.30	44	ADEEIAALV VDNGSGMCKVAPEEHPVLLT EAP LNPKTTGIVMDSGDGV THTVPIYEGY ALPHAILRDLT DYL MKGYSFTTTAERLCYV ALDFEQEMAT AASSSSLE KS YELPDGQVITIGNERDLYANTVL SGGTTMYPGIAD REITALAPSTMKQEYDESGPSIVHR
303	Actin-related protein T2	ACTRT2	574	42400/5.48	27	AGLSGEIGPRFKTPLTGANQKKYFVGE EALHRGLITGWEDMEKHLFEWELGVKANDQPVLMT EPSLNPRDITEHLTRALVDDIKEKQMWVTSADFKEFGTSVIQR
308	-	-	213	42400/5.48	9	YFVGE EALHRHLFEWELGVKANDQPVLMT EPSLNPR
318	-	-	208	42400/5.48	12	AGLSGEIGPRKYFVGEEALHRAN DQPVLMTEPSLNPRALVDDIKEK
438	F-actin-capping protein subunit beta	CAPZB	803	34176/6.02	52	VGTADYGGASDQSDQQLDCALDLMR RLPPQQIEKDYLLCDYNRSPWSNKYDPPLEDGAMPSAR KLEVEANNAFDQYRIKGCWDSIHVVEVQEKLTSTVM LWLQTNKSGSGTMNLGGSLTRQMEKDE TVSDCSPHIANIGRSTLNEIYFGKNDLVEALKR
456	Acrosin-binding protein	ACRBP	143	61641/4.82	5	HLAACSLCDFCSLKFYGLDLYGGLRMDFWCAR
459	-	-	204	61641/4.82	9	RHLAACSLCDFCSLKFYGLDLYGGL RMDFWCARICDTEYVQYPNYCAFK
489	-	-	130	61641/4.82	5	HLAACSLCDFCSLKFYGLDLYGGLRMDFWCAR
671	-	-	69	62303/5.28	1	FFALLTPTWK
935	-	-	166	61641/4.82	8	HLAACSLCDFCSLKFYGLDLYGG LRMDFWCARICDTEYVQYPNYCAFK
501	Izumo sperm-egg fusion protein 4	IZUMO4	129	25691/5.91	11	ELHLAIPAEITREQVHLIQNAIIESR
573	-	-	130	25691/5.91	11	ELHLAIPAEITREQVHLIQNAIIESR
625	Cadherin-1	CDH1	69	98618/4.76	1	VSFEGCAGLPR
649	Sperm acrosome membrane-associated protein 3	SPACA3	104	18543/5.87	20	VLQDFGLEGYRNLNPNVPNLCQMYCSDLLNPNLK
663	-	-	66	18543/5.87	6	VLQDFGLEGYR
670	Sperm acrosome-associated protein 5	SPACA5	54	18024/5.55	6	HILDDIMCAK

These proteins were separated into distinct categories: binding proteins sperm-egg fusion, acrosomal membrane proteins, metabolic enzymes, heat shock proteins, and cytoskeletal and proteins involved with spermatozoa motility. The functions of these proteins were described according to the SwissProt database.

**Figure 2:**
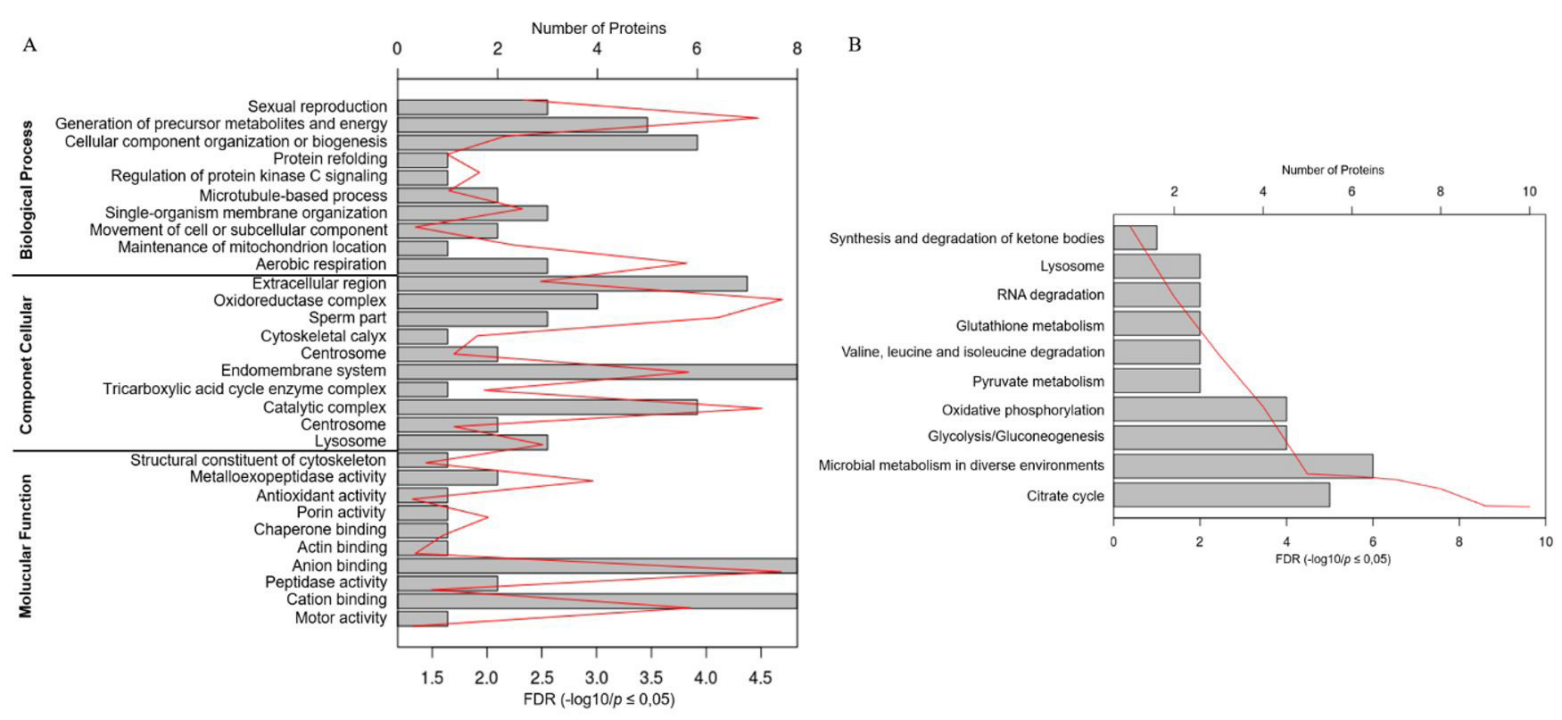
Representative Gene Ontology terms (A) and metabolic pathways (B); both resulted from an enrichment based on the proteins identified in the Saanen goat spermatozoa using data from Bos taurus in the Gene Ontology and KEGG Pathway analyses. The three subclassifications of GO were considered: biological process, cellular component and molecular function. We considered only the GO terms and metabolic pathways with false discovery rate (FDR) values ≤ 0.05.

**Figure 3:**
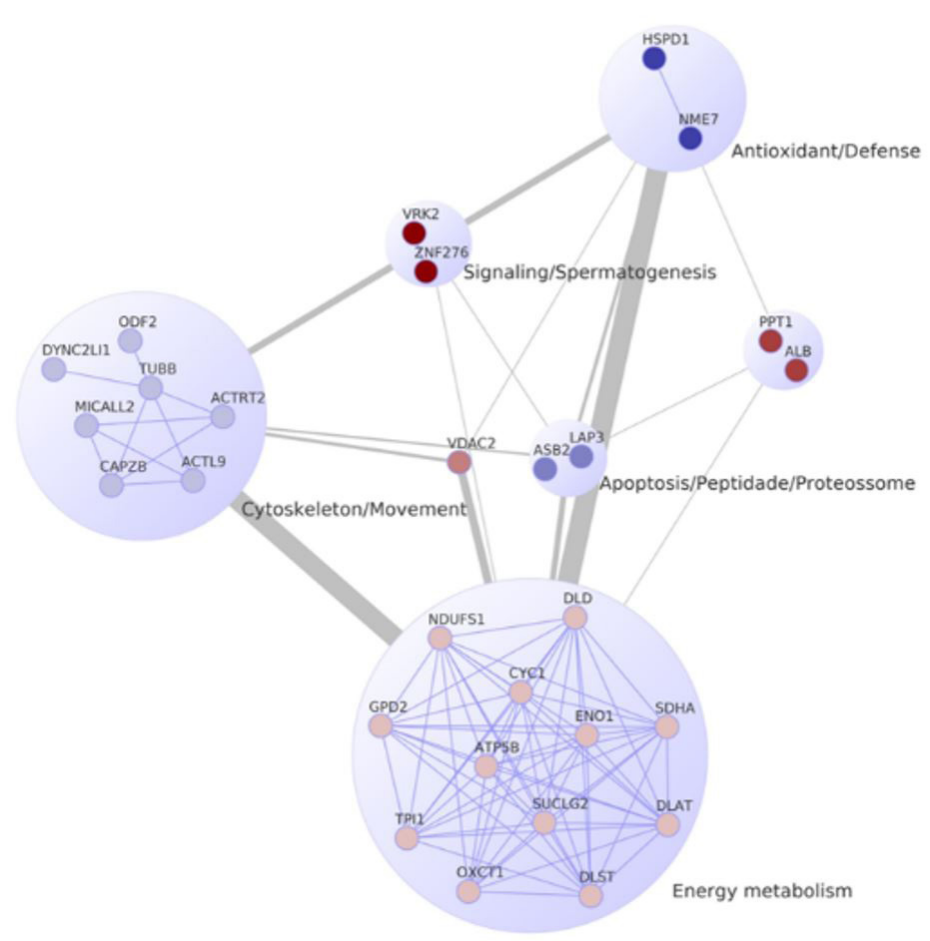
Protein-protein interaction network (PPI) based on information for Bos taurus containing the proteins identified for the Saanen breed. The functions or processes performed by proteins are highlighted in the modules. The pathways enriched with FDR values ≤ 0.05 and a confidence threshold of 0.400 in the network were considered.

Furthermore, the identified proteins were submitted to enrichment analysis to discover their functions based on the terms GO and KEGG Pathway. Figure 2 shows the main ontologies and metabolic pathways identified for the proteins obtained from the Saanen spermatozoa. Among the functions and pathways, the following stand out: aerobic respiration, cytoskeletal constituents/cellular movement, and the regulation of signalling by protein kinases. In an attempt to reduce the terms and functions identified, it was possible to construct a protein-protein interaction network highlighting functional modules ( Figure 3 ). It was possible to visualize two main modules in the proteins considered in the network, 42.85% were related to energetic metabolism, followed by 25% to the constituents of the cytoskeleton and movement.

## Discussion

Spermatozoa proteomics studies are important because significant changes occur during maturation and capacitation to confer fertility capacity to sperm cells ( Brewis and Gadella, 2010 ). With the apparent absence of gene transcription, sperm cell functionality is largely dependent on post-translational modifications, which are evidenced in the processes of epididymal maturation and capacity building ( Aitken and Baker, 2008 ). Thus, the study of the sperm proteome is critical to understanding the role of proteins in animal reproductive physiology.

From this perspective, this study provides relevant molecular data about the Saanen proteomic profile of spermatozoa associated with reproduction, contributing to its molecular information. The distribution of spots by pI and MW in two-dimensional gels reflects the protein diversity of the spermatozoa.

The analysed spots are more widely distributed in the pI range of 5-6 and have a molecular mass between 20-80 kDa, confirming the results obtained by Matos (2012) , who studied sperm proteins in Moxotó goats. Similar results were obtained with horse spermatozoa ( Dias, 2006 ).

Among the proteins identified by mass spectrometry, thirty-one are involved in reproductive processes. These proteins were separated into distinct categories: binding proteins sperm-egg fusion, acrosomal membrane proteins, metabolic enzymes, heat shock proteins and cytoskeletal proteins.

Binding proteins have been described in the literature in combination with the spermatozoan surface. Spots 501 and 573 were identified as protein Izumo Sperm-Egg Fusion, whose function is to mediate the interaction of the spermatozoa with the membrane of the egg. Izumo is a spermatic membrane protein belonging to the Superfamily immunoglobulins and is involved in cell adhesion and interaction ( Lorenzetti et al., 2014 ). Izumo acts together with an oocyte receptor, Juno, in the membrane fusion process ( Klinovska et al., 2014 ). In bovine spermatozoa, Izumo 1 and 4 demonstrated success in fertility ( Byrne et al., 2012 ). In studies with mice, the presence of both protein and its receptor, therefore, was demonstrated to be essential for fertilization ( Bianchi et al., 2014 ).

Acrosin binding protein (spots 456, 459, 489, 671, and 935)  is a calcium-dependent phosphoprotein and is located in the acrosomes of the germ cells of several species. This protein is involved in the condensation of zymogen in the acrosomal matrix and in sperm capacitation ( Dubé et al., 2005 ; Vilagran et al., 2013 ). In addition, it is an important regulator of proteolytic processing events during the disassembly of the acrosomal matrix ( Foster 2013 ). Guyonnet et al. (2011) examined rat spermatozoa by indirect immunofluorescence and identified the location and function of the protein in the acrosomal matrix. In goats, van Tilburg et al. (2015) detected and elucidated its reproductive function in males by mass spectrometry. According to Kim et al. (2015a) , acrosin binding protein can be used as an indicator for the sexual maturation of stallions as well as to monitor normal spermatogenesis in testicular tissues or the development of germ cells in vitro.

Cadherin-1 (spot 625) is involved in the mechanisms that regulate cell-cell adhesion, motility and the proliferation of epithelial cells. Lie et al. (2011) described it as responsible for cell adhesion and as an essential component in the basal part of the blood-testis barrier. Vazquez et al. (2013) compared human spermatozoa and observed that a decrease in the percentage of immunoreactive E-cadherin was associated with lower fertility performance. Thus, they proposed that this protein could be a structural and functional biomarker associated with fertile spermatozoa.

Sperm surface membrane protein 3 (spot 649, 663) and sperm surface membrane protein 5 (spot 670) are involved in the adhesion of spermatozoa to the egg and its fusion with the egg during fertilization. According to Nixon et al. (2007) , these proteins perform such functions by forming a glycoprotein receptor in the equatorial segment and binding to the N-acetylglucosamine residue; these proteins are also essential for spermatozoan-oocyte fusion.

Dihydrolipoyl mitochondrial dehydrogenase (spots 187 and 198) is a protein with catalytic activity that acts in the lipoamide dehydrogenase glycine cleavage system and in the dehydrogenase complex of alpha-keto acid. Both are involved in increasing motility during sperm capacitation and the acrosome reaction. Panneerdoss et al. (2012) studied the proteins involvement in the hamster spermatozoa lactate metabolism, and they induced the inhibition of this protein; this resulted in the accumulation of lactate and lead to a reduction in the intracellular pH and calcium levels, which ultimately blocked the capacity of the spermatic and acrosome reactions.

HSPD1 or HSP60, spots (174 and 181), has the function of protecting spermatozoa from degradation due to heat stress. Dangi et al. (2012) used goat blood to analyse HSP60 expression in winter and summer and noted a significant increase with the increase in temperature. In another study, Pei et al. (2012) induced thermal stress in rabbit testes for nine weeks and observed a significant increase in its expression levels, suggesting that HSP60 plays a multifunctional protective role in the testis during thermal shock.

F-actin capping protein (spot 438) is associated with the cytoskeleton and binds independently to Ca ^2+^ for the growth of actin filaments. Moreover, actin filaments are associated with the spermatozoa through the calcium channel and are involved in the process of fertilization and increasing motility ( Baker et al., 2010 ).

The actin family proteins also participate in cytoskeletal organization. Among them, actin-like protein 9 (spot 291), actin cytoplasmic type 5 (spot 307) and actin-related protein T2 (spots 303, 308 and 318) were identified. The specific synthesis of actin family proteins in the testes occurs at the end of spermatid differentiation. They were studied in bovine ( Byrne et al., 2012 ) and ovine ( van Tilburg et al., 2013 ) spermatozoa.

Among the proteins identified, α- and β-tubulin and outer dense fibre protein 2 are related to spermatozoa motility. The α-tubulin (spots 178 and 274) and β-tubulin alpha-3C/D chain (spots 401, 421, 498, 577 and 748) are the majority constituents of microtubules connected to two GTPs, which are responsible for producing energy. Another specific function is the regulation of spermatogenesis and the adaptation of the cytoskeleton to ensure movements ( Sperry, 2012 ). α-Tubulin and β-tubulin have various post-translational modifications, including acetylation. According to Bhagwat et al. (2014) , the acetylation of these proteins is associated with sperm motility.

Outer dense fibre protein 2 (spots 235, 236 and 325) is a spermatozoa tail component that influences modulation and spermatozoa motility. According to Hashemitabar et al. (2015) , this protein had lower levels of expression in asthenozoospermic patients, causing abnormalities in the external dense fibres and reductions in the elasticity of the sperm’s flagellum, affecting sperm motility. Saram et al. (2015) demonstrated the proliferation of cells and changes in the expression of this protein during the process of ciliogenesis. Chung et al. (2014) used specific antibodies to mark this protein, determining its distribution in the scourge through 3D structures.

The proteomic profile of Saanen goat spermatozoa was established and identified important proteins involved in the reproductive process. Among them, the proteins with major coverage were involved in spermatogenesis and motility. Our data provide a better understanding of the proteins involved in the reproductive physiology of goats and for molecular studies that contribute to the elucidation of fertility processes and the improvement of animal reproduction.

## Abbreviations

3-[(3-Cholamidopropyl)dimethylammonio]-1-propanesulfonate: CHAPS.

Bovine serum albumin: BSA.

Coomassie Brilliant Blue: CBB.

Dithiothreitol: DTT.

Immobilized pH Gradient: IPG.

Mass spectrometry: MS.

One-dimensional electrophoresis: 1-DE.

Polyacrylamide gel electrophoresis: SDS-PAGE.

Polyethylene glycol p-(1,1,3,3-tetramethylbutyl)-phenyl ether: Triton X-100.

Sodium dodecyl sulfate: SDS.

Tetramethylethylenediamine: TEMED.

Tricarboxylic acid: TCA.

Two-dimensional electrophoresis: 2-DE.

## Annex 1 Volume by collection and the quantification of the total sperm proteins of Saanen goats using the Bradford method.

**Table:**

**Sample**	**Volume (mL)**	**Protein Concentration (μg/μl) **
Animal A (378)	1,33±0,24a	25,95
Animal B (310)	1,73±0,39a	18,85
Animal C (250)	1,67±0,44a	22,2
Animal D (238)	1,03±0,20a	28,6
Animal E (910)	0,90±0,06a	14,25
